# Statistical analysis plan for the Urodynamics for Prostate Surgery Trial; Randomised Evaluation of Assessment Methods (UPSTREAM)

**DOI:** 10.1186/s13063-017-2206-y

**Published:** 2017-10-03

**Authors:** Grace J. Young, Amanda L. Lewis, J. Athene Lane, Helen L. Winton, Marcus J. Drake, Peter S. Blair

**Affiliations:** 1 Population Health Sciences, Bristol Medical School, University of Bristol, Canynge Hall, 39 Whatley Road, Bristol, BS8 2PS UK; 2 Bristol Randomised Trials Collaboration (BRTC), University of Bristol, Canynge Hall, 39 Whatley Road, Bristol, BS8 2PS UK; 3 North Bristol NHS Trust, Bristol Urological Institute, Level 3, Learning and Research Building, Southmead Hospital, Bristol, BS10 5NB UK; 4 Translational Health Sciences, Bristol Medical School, University of Bristol, Bristol, UK

**Keywords:** Statistical analysis plan, Randomised controlled trial, Urodynamics, Lower urinary tract symptoms, Non-inferiority trial

## Abstract

**Background:**

Current management for men with lower urinary tract symptoms (LUTS) is a pathway that results in prostate surgery in a significant proportion. While helpful in relieving benign prostatic obstruction (BPO), surgery may be ineffective for men suffering from difficulties not relating to BPO. The UPSTREAM trial started recruitment in October 2014 with the aim of establishing whether a care pathway including urodynamics (a diagnostic tool for BPO and thus an indication of whether surgery is needed) is no worse for men, in terms of symptomatic outcome, than one without (routine care).

**Methods/design:**

This analysis plan outlines the main outcomes of the study and specific design choices, such as non-inferiority margins. The trial is currently recruiting in 26 hospitals across the UK, randomising men to either urodynamics or routine care, with recruitment set to end on the 31 December 2016. All outcomes will be measured 18 months after randomisation to allow sufficient time for surgical procedures and recovery. The primary outcome is based on a non-inferiority design with a margin of 1 point on the International Prostate Symptom Score (IPSS) scale. The key secondary outcome for this trial is surgery rate per arm, which is estimated to be at least 18% lower in the urodynamics arm. Surgery rates, adverse events, flow rate, urinary symptoms and sexual symptoms are secondary outcomes to be assessed for superiority. This is an update to the UPSTREAM protocol, which has already been published in this journal.

**Discussion:**

This *a priori* statistical analysis plan aims to reduce reporting bias by allowing access to the trial’s objectives and plans in advance of recruitment end. The results of the trial are expected to be published soon after the trial end date of 30 September 2018.

**Trial registration:**

ISRCTN registry, ISRCTN56164274. Registered on 8 April 2014.

## Introduction

The Urodynamics for Prostate Surgery Trial (UPSTREAM) is a two-arm trial, which randomises men with bothersome lower urinary tract symptoms (LUTS), for whom surgeons would consider offering surgery, between two treatment pathways. The intervention arm is a slightly invasive care pathway to see if surgery is needed based on urodynamic tests with multichannel cystometry while the control arm is a care pathway without urodynamics tests (current routine care). The UPSTREAM trial is a pragmatic, randomised controlled trial with a non-inferiority primary outcome. More details concerning the trial’s rationale can be found in the published protocol [1]. Briefly, the primary aim of the UPSTREAM trial is to establish whether a treatment pathway for LUTS that includes urodynamics is no worse for men, in terms of symptom burden, than current routine care (without urodynamics).

Research suggests that the majority (90%) of men aged 50 to 80 years suffer from at least one lower urinary tract symptom. These symptoms increase with age and relate to a spectrum of urinary problems both in the storage phase (increased daytime urinary frequency, nocturia, urgency, incontinence) and the voiding phase (slow stream, intermittency, hesitancy, straining and dribbling) [2]. Voiding LUTS may be caused by either bladder dysfunction caused by poor expulsion strength of bladder muscle or benign prostate obstruction (BPO). Prostate surgery such as transurethral resection of the prostate (TURP) is an invasive procedure which sometimes causes unpleasant adverse events, such as incontinence and difficulties with sexual function [1]. A large proportion of men suffering from BPO have improved LUTS after surgery; however, for those with bladder dysfunction this procedure is deemed unnecessary and potentially harmful [1]. Estimates from hospital audit data suggest that between 18% and 28% of the men currently undergoing prostate surgery for LUTS do not have BPO; such men are at risk of undergoing potentially unnecessary surgery [1].

Urodynamics is a diagnostic test that can ascertain whether BPO is present and therefore indicate whether the patient may benefit from surgery or not [3]. It is anticipated that use of this diagnostic tool will reduce surgery rates (the key secondary outcome) and this, along with the primary outcome, International Prostate Symptom Score (IPSS) score, will be measured 18 months after randomisation. The trial will cover many aspects of the men’s treatment and recovery with a clinical, cost-effectiveness and qualitative analysis. If the intervention group yield a similar symptom burden to those under usual care and the surgery rates are reduced then this may warrant consideration of the test becoming a more prominent feature in the assessment pathway for men with LUTS.

This analysis plan was written and finalised by the trial team during the recruitment period for UPSTREAM. Original drafts began in January 2015 with a version finalised in September 2016. Content has been approved by the Independent Data Monitoring Committee (IDMC) and Trial Steering Committee (TSC) chairs. Although one DMC report had already been created and presented prior to this final version, no 18-month data were available and all statisticians and members of the DMC remained blinded to the trial arm allocation. No formal statistical analyses have been conducted thus far.

## Methods/design

### The IPSS score

The primary outcome will be measured using the IPSS, originally known as the American Urological Association symptom index for benign prostatic hyperplasia [4]. The scale ranges from 0 to 35, where higher scores indicate more severe symptoms. It comprises seven sections scored on a scale of 0 to 5 with 0 referring to “not at all” and 5 referring to “almost always”. These seven sections are “incomplete emptying”, “frequency”, “intermittency”, “urgency”, “weak stream”, “straining” and “nocturia”. To put this into perspective a recent study found men with voiding symptoms, storage symptoms and no symptoms to have IPSS scores of 16.8, 14.6 and 8.5, respectively [5]. The IPSS questionnaire also includes a quality of life (QoL) measure which asks how patients would feel if their urinary conditions remained the same for the rest of their life. This ranges from 0 to 6 where 0 is “delighted” and 6 is “terrible”. The IPSS questionnaire is used at baseline, 6, 12 and 18 months. For the sensitivity analysis, if men are missing a baseline questionnaire, 6-month scores may substitute baseline scores if men have not received treatment before 6 months. If men are missing 18-month scores then the 12-month scores may substitute 18-month scores if men have received all allocated treatment by this time point.

### Objective

This trial investigates the research question: “Is a care pathway including urodynamics no worse for men, in terms of symptom outcome, than routine care (without urodynamics) at 18 months after randomisation”. The null hypothesis is that the routine care is superior to urodynamics while the alternative hypothesis is that urodynamics is non-inferior to routine care. The IPSS score will be used to calculate this:

$$ {\displaystyle \begin{array}{l}{\mathrm{H}}_0:{\upmu}_{\mathrm{routine}\  \mathrm{care}}\hbox{--} {\upmu}_{\mathrm{urodynamics}}\le \hbox{-} 1\\ {}{\mathrm{H}}_1:{\upmu}_{\mathrm{routine}\  \mathrm{care}}\hbox{--} {\upmu}_{\mathrm{urodynamics}}>\hbox{-} 1\end{array}} $$

For example, if the mean IPSS score for the urodynamics arm is 17 at 18 months and the mean score for the routine care arm is 15, the urodynamics arm will be considered inferior. Conversely, if the IPSS score for the urodynamics and routine care arms were 17.5 and 17, respectively, then urodynamics would be considered non-inferior. As a main secondary outcome it will also establish whether “inclusion of urodynamics reduces rates of bladder outlet surgery, compared with routine care”. All analyses will be based on the questionnaire and consultation data collected at the 18-month follow-up consultation. This time frame should allow enough time for the urodynamics procedure, transurethral resection of the prostate, additional treatments and recovery from these procedures.

### Non-inferiority margin

Non-inferiority trials are particularly helpful when you are testing whether a new treatment is no worse than the current routine treatment by more than an acceptable amount. This is often the case when investigators believe that the new treatment may offer other advantages over the comparator treatment, e.g. safety, costs [6]. To fully appreciate the reasoning behind choosing to conduct a non-inferiority trial it is important to understand the research questions behind the primary and secondary outcomes. The key secondary outcome will be the proportion of men who have surgery. In the event that this is reduced, as anticipated in the urodynamics arm, this could reduce unnecessary surgical interventions for men who are suffering from LUTS which is not attributable to BPO. Should this be the case, it is then important to establish whether this change does not worsen patient symptoms. With this in mind, a non-inferiority approach was chosen to establish whether including urodynamics leads to outcomes which are not inferior (rather than equivalent) to a pathway without urodynamics. For the primary outcome, a difference in LUTS score of 1 point (on the IPSS scale) was considered non-inferior. The trial team felt that a 1 point non-inferiority margin was appropriate with the following justification:

- A difference of 3 points and 0.5 points on the total IPSS score and QoL IPSS score, respectively, indicates a minimally clinical important difference (MCID) for the overall urinary condition [7]. While this is the case for the overall IPSS score, a difference of < 3 may involve substantial changes in symptom bother associated with a certain subscale [8], especially in relation to storage-type LUTS. Given this MCID the team felt that a substantial yet conservative estimate was needed below this figure.
- One void per night does not generally prove a problem for patients whereas two or more is considered substantially “bothersome” by most patients [9]. Given that a 1-point difference on the IPSS scale could indicate a difference in nocturia of 2 to 1 the team considered this to be a significant turning point on the IPSS scale.
- The trial team felt that a 1-point difference was a conservative estimate and, given this, would avoid false claims of non-inferiority. This margin will be explored using the bothersome measures in the International Consultation on Incontinence Modular Questionnaire Male Lower Urinary Tract Symptoms (ICIQ-MLUTS) [10].

### Sample size calculation

The sample size calculation was constructed from both the primary and key secondary outcomes. For the primary outcome, IPSS score at 18 months, the trial needed to detect a non-inferiority margin of 1 point. Using STATA v14.1 [11], calculating a one-sided *t* test with a common standard deviation of 5, mean difference of 1, 80% power and 5% alpha level, the required sample size was 310 men per arm. It was estimated that 20% attrition would occur due to withdrawals and losses to follow up. Adjusting for this led to an increased sample size of 388 per arm, 776 in total.

The key secondary outcome of surgery rate per arm was a superiority hypothesis where the team had anticipated a difference in surgery rates between the arms. Data from hospital audit indicated that 73–83% of men presenting with LUTS were having surgery [1]. Had the urodynamics test been conducted on the same men, the data indicate that only 60% would have had surgery based on the prevalence of impaired bladder contractility contraindicating surgery. In order to show an absolute risk reduction in surgery from 73% to 60% in the intervention arm (relative risk reduction = 18%), a total of 291 men would be needed in each group. This calculation was based on a two-sided 5% significance level and 90% power. Inflating the sample size to account for 20% attrition increased the sample size to 364 per arm, 728 in total. Thus to have sufficient power for both hypotheses the trial aimed to recruit 800 men in total, around 400 men per arm.

### Baseline characteristics

Baseline characteristics will be compared between the two arms by reporting relevant summary statistics in order to determine whether any potentially influential imbalance occurred, by chance, between the two arms. Characteristics will be reported as means (SD), medians (IQR) or proportions (percentage) depending on the nature and distribution of the data. *P* values will not be reported for differences between the two groups at baseline, since appropriate randomisation methods will have accounted for this. Therefore, any differences identified would be due to chance such that a significant *p* value would in reality be representative of a type 1 error (a rejection of the null hypothesis of no relationship when it is in fact true). Large differences at baseline (more than half a SD for continuous variables or 10% for categorical variables) will be investigated in a sensitivity analysis.

The following baseline measures will be recorded and compared: age, centre, ethnicity, Index of Multiple Deprivation (based on postcode), comorbidities, digital rectal examination (DRE) findings (e.g. benign enlargement), maximum flow rate (ml/s), post void residual volume (ml), voided volume (ml), additional tests (e.g. cystoscopy), IPSS and QoL score, ICIQ-MLUTS and International Consultation on Incontinence Questionnaire - Sexual Matters associated with Lower Urinary Tract Symptoms (ICIQ-MLUTSsex).

### Primary analyses

The primary analysis will be conducted under the intention to treat (ITT) principle using a linear regression model. To test the robustness of the results we will use the per-protocol principle in a sensitivity analysis. The dependent variable will be the overall IPSS at 18 months post randomisation. The tested null hypothesis is that a treatment pathway with urodynamics is inferior to a treatment pathway without urodynamics (routine care). The mean difference will be presented with the 95% confidence interval, adjusted for the baseline IPSS score and centre. The difference will be calculated as the mean IPSS score in routine care minus the mean IPSS score in urodynamics. After adjustment for centre and baseline IPSS score, urodynamics will be classed as non-inferior if the lower band of the 95% confidence interval lies above minus one. Should the lower confidence interval band exceed both minus one and zero then we may test whether urodynamics is superior to routine care, as testing for non-inferiority before superiority does not require a statistical penalty for multiple testing [12].

### Secondary analyses

Similar to the primary analysis model, all secondary analyses will be on an intention to treat (ITT) basis, adjusting for centre and baseline measures (if applicable) for the following outcomes:

1. Surgery rate at 18 months (key secondary): using logistic regression, we will be estimating the difference in surgery rates between the groups. It was hypothesised that the urodynamics procedure would reduce “unnecessary” further intervention and therefore surgery rate, in the intervention arm.
2. Adverse events due to testing and/or treatment at 18 months: all adverse events will be compared between the groups at 18 months; including treatment-related and unrelated adverse events and deaths. The number of cases of acute urinary retention will also be examined. These will be analysed according to the number of events altogether and the number of events per patient. Logistic and ordinal logistic regression will be used to compare the groups.
3. Measures from the ICIQ [10] (ICIQ-MLUTS, ICIQ-MLUTSsex) and IPSS QoL at 18 months: alongside the IPSS score, the IPSS QoL and ICIQ measures will offer a more detailed analysis of LUTS in terms of severity, bother and impact on quality of life. We will calculate voiding and incontinence scores along with the proportion of men with a high frequency of voids during the day and night. We will also look at the proportion of men with sexual dysfunction and the effects this has on their lives. Linear and logistic regression will be used for continuous and binary outcomes respectively, adjusting for centre and respective baseline measures. Non-parametric techniques may be employed if model assumptions are not met.
4. Maximum urinary flow rate (Qmax) at 18 months: the urinary flow will be measured at the 18-month follow-up clinic appointment and analysed using linear regression, adjusting for centre and baseline Qmax. Adjusted for baseline measures this will give an indication of how well a man’s urinary symptoms have improved (or worsened), at the end of their treatment follow up.

There will be additional cost-effectiveness and qualitative analysis outcomes not mentioned in this statistical analysis plan that will present measures on QoL.

### Sensitivity analyses

Sensitivity analyses will be utilised to ensure that the primary analysis model results are robust to appropriate adjustments and imputations. Pre-specified sensitivity analyses include:

1. Per-protocol analysis: the per-protocol analysis allows assessment of treatment effect among those who received the treatment that they were assigned to. This analysis will include patients who received the treatment they were assigned to (compliers). Therefore, from those randomised to urodynamics, if a patient did not receive the procedure they will be removed from this analysis. Patients who received urodynamics despite being randomised to the routine-care arm will also be excluded. For non-inferiority trials it is recommended that both ITT and per-protocol analyses are reported [13].
2. Complier average causal effect (CACE) analysis: the CACE analysis allows unbiased assessment of treatment effect, after separating the intervention arm into compliers and non-compliers. Alternative approaches may be considered if the contamination rate is high [14].
3. Mixed-effects model: through the reviewing process of this analysis plan, it was strongly recommended that the team use a mixed effects repeated measures model (MMRM). The trial team anticipates that missing data will be missing at random (MAR) and therefore feel that MMRM is appropriate to evaluate the difference in treatments, while accounting for missing IPSS scores [15].
4. Imputation using 6 and 12 month scores: for those with missing baseline scores, the 6-month IPSS score may be used as a substitute unless any treatment has been received prior to the 6-month questionnaire. Similarly, for those with missing 18-month scores, the 12-month IPSS score may be used as a substitute. If the primary outcome has more than 15% missing data (which could benefit from supplementation from 6 and 12 month data) we may consider this model for our main primary analysis.
5. Imputation for missing data: missing data for the primary outcome, assumed to be MAR will be imputed under conservative assumptions and the effect of missing data investigated. If the data, as anticipated, is MAR the trial team will consider an approach such as multiple imputation by chained equations (MICE). In the event that the data appear to be missing not at random (MNAR), then alternative approaches, such as pattern mixture model (PMM) will be adopted. The handling of missing data will follow the principles specified in the European Medicines Agency guidelines [16] and any changes to the methods described here will be fully justified in the study report and publication. For the imputation model adopted, a pre-specified random seed of 648 has been chosen.
6. Adjustment for clinically important confounders: the primary analysis and key secondary analysis will be adjusted for centre, age, comorbidities and symptom severity (all collected at baseline) that were pre-specified as clinically important by the investigators.
7. Adjustment for imbalance at baseline between the arms: to ensure that the groups are balanced in terms of unfavourable baseline characteristics, we will adjust for any imbalance at baseline. A difference of 0.5 SD or 10% between the descriptive data will be considered an imbalance.
8. Adjustment for time between surgery and the 18 month time point: the primary outcome - IPSS score at 18 months - is measured 18 months after randomisation. It was originally hoped that this would allow a 6-month “post-surgery” gap to allow us to see the long-term effects of surgery. However, given the nature of surgery waiting lists we will be adjusting for “time since surgery”’ as a sensitivity analysis to avoid spurious results caused by symptoms retained from recent surgery.

If any of the sensitivity analyses prove important they may be used for the secondary outcomes.

### Subgroup analyses

Formal tests of interaction between the dichotomised variables and treatment pathway will be carried out to test whether treatment effect differ between different patient groups. Interaction tests are likely to be underpowered, therefore emphasis will be placed on the point estimates and confidence intervals generated, rather than any associated *p* values. These will be at the 10% alpha level and interpreted as hypothesis generating, as any formal testing will be unreliable. They will be applied to the primary analysis (IPSS score) and the main secondary analysis (surgery rates), including (but not limited to):

1. Age (above and below the median)
2. Flow rate (>12 ml/s vs. ≤12 ml/s)
3. Maximum voided volume (<200 ml vs. ≥200 ml), measured in the baseline bladder diary
4. Storage dysfunction: nocturia (yes vs. no)
5. Severity of storage LUTS (more substantial vs. less substantial)

### Presentation of figures and tables

When publishing the trial results, Figure 1 will depict the trial flow (Consolidated Standards of Reporting Trials (CONSORT)) diagram, identifying the numbers of patients who were ineligible, who declined, withdrew, were lost to follow up or were excluded from the analysis (e.g. due to missing questionnaire data). Table 1 will offer proportions (percentage), means (SD) or medians (IQR) of the baseline covariates for those in the UPSTREAM trial, by arm. If adequate data are collected we will also compare baseline characteristics between those who entered the study and those who declined or were ineligible. The primary, secondary, sensitivity and subgroup analyses will all be presented in tables with difference estimates, confidence intervals and *p* values. It is anticipated that different centres may interpret urodynamic results differently, potentially affecting surgery rates. Therefore, estimates will be adjusted for the baseline measure in the question and the centre. Adverse events will be differentiated by arm and by relation to treatment, and graded using the Clavien-Dindo [17] classification for surgery-based events. An independent reviewer will assess the assigned relation to treatment and the Clavien-Dindo scoring of surgery-related adverse events.

## Discussion

Following on from the protocol this paper aims to add a layer of specificity to the UPSTREAM trial, to prevent biased and misleading statistical inferences. However, in the event that the analytical tests we have pre-specified are not appropriate for the final data, then alternative approaches may be considered and full justification given.

When UPSTREAM comes to publication, an unbiased and informed overview of pathways for men with LUTS should result. In order to do this it is necessary to be sure that the conclusions drawn are robust and not due to statistical multiplicity. The trial team has defined, a priori, both a primary and key secondary outcome on which they based their sample size. We are not powered to test our secondary analyses and will therefore interpret any *p* values with due caution. This field of medicine is relatively unexplored, offering numerous but potentially sceptical observers, therefore the primary analysis details have been set out in advance of recruitment end.

### Differences between the protocol and the statistical analysis plan

This analysis plan is very similar to the protocol published in December last year with only a few minor changes. In the protocol abstract we stated that the aim of the trial was to “determine whether a care pathway not including invasive urodynamics is no worse for men in terms of symptom outcome than one in which it is included”, at 18 months after randomisation. Given that this a non-inferiority trial and urodynamics is the intervention we are testing, this should have read “determine whether a care pathway including invasive urodynamics is no worse for men in terms of symptom outcome than one in which it is not included”. However, our non-inferiority margin has not changed and we are still classing a one-point difference on the IPSS scale as non-inferior, as mentioned in the “Statistics and data analysis” section of the published trial protocol.

Originally in the protocol paper it was stated that randomisation would be stratified by centre. UPSTREAM in fact, uses a “simple randomisation”’ approach whereby centres utilise an automated web/telephone randomisation system provided by the Bristol Randomised Trials Collaboration (BRTC). Since publishing the protocol paper the trial has also received funding for a 6-month extension, which will now mean that recruitment will end in December and the trial will officially end in September 2018.

Analysis of primary and secondary outcomes remains unchanged. However, the subgroup analyses have been updated and some sensitivity analyses added. Originally, we had included factors that could only be found in the urodynamics arm (e.g. whether or not the patient was suffering from BPO). After careful consideration these were moved to exploratory analyses. All subgroups listed in this update can be found in both arms of the study and will therefore be used in an interaction term in the primary and key secondary analyses.

### Trial status

This manuscript was submitted before recruitment ended (14 December 2016) and underwent minor revisions, based on reviewers’ feedback, after recruitment had ceased (23 of May 2017).

## Abbreviations

BPO: Benign prostatic obstruction
BRTC: Bristol Randomised Trials Collaboration
CACE: Complier average causal effect
CONSORT: Consolidated Standards of Reporting Trials
DRE: Digital rectal examination
ICIQ: International Consultation on Incontinence Questionnaire
ICIQ-MLUTS: International Consultation on Incontinence Questionnaire – Male Lower Urinary Tract Symptoms
ICIQ-MLUTSsex: International Consultation on Incontinence Questionnaire – Sexual Matters associated with Male Lower Urinary Tract Symptoms
IDMC: Independent Data Monitoring Committee
IPSS: International Prostate Symptom Score
IQR: Interquartile range
ITT: Intention to treat
LUTS: Lower urinary tract symptoms
MAR: Missing at random
MCID: Minimally clinically important difference
MICE: Multiple imputation by chained equations
MMRM: Mixed-effects model for repeated measures
MNAR: Missing not at random
PMM: Pattern mixture model
Qmax: Maximum urinary flow rate
QoL: Quality of life
SD: Standard deviation
TSC: Trial Steering Committee
TURP: Transurethral resection of the prostate
UK: United Kingdom
UPSTREAM: Urodynamics for Prostate Surgery Trial: Randomised Evaluation of Assessment Methods
